# Management Strategy for Non-Responsive and Refractory Celiac Disease in Adults: A Review Article

**DOI:** 10.3390/jcm14196934

**Published:** 2025-09-30

**Authors:** A. Al-Toma

**Affiliations:** Department of Gastroenterology and Hepatology, St. Antonius Hospital, 3430 EM Nieuwegein, The Netherlands; a.altoma@antoniusziekenhuis.nl

**Keywords:** non-responsive celiac disease, refractory celiac disease, gluten-free diet, adult celiac disease, treatment, management, review

## Abstract

**Background/Objectives:** A substantial number of adults with celiac disease (CeD) experience ongoing symptoms despite consuming a gluten-free diet (GFD), a condition labelled as non-responsive CeD (NRCD). However, many experts contest the term, viewing NRCD not as a distinct entity, but as a clinical prompt to identify a specific underlying cause. A minority develop refractory CeD (RCD), a severe complication with persistent villous atrophy, after beginning a diet excluding gluten exposure. This review synthesizes evidence to provide a practical, stepwise algorithm for managing these complex patients. **Methods:** A narrative review was conducted based on a targeted literature search of major databases seeking studies on adults with NRCD or RCD, focusing on diagnostic and therapeutic strategies. **Results:** The most frequent cause of NRCD is inadvertent gluten ingestion. Objective and systematic assessment, including expert dietitian evaluation and testing with gluten immunogenic peptides (GIPs) in stool or urine GIP testing, is essential before the investigation seeking to exclude or establish RCD. This is a critical step for evaluating adherence beyond the patient self-report. The management of confirmed RCD hinges on precise subtyping via duodenal biopsy with immunophenotyping. While RCD type I (RCD-I) typically responds to budesonide, RCD type II (RCD-II) carries a high risk of lymphoma and necessitates aggressive therapies in specialized centers. **Conclusions:** This review underscores the necessity of a structured, hierarchical diagnostic approach in distinguishing persistent gluten exposure from true RCD. The integration of GIP testing and specialist dietitian review is a cornerstone of modern management. The findings highlight significant evidence gaps, particularly for RCD-II, and aim to guide clinical practice and inform future research towards standardized protocols.

## 1. Introduction

Celiac disease (CeD) is a chronic, immune-mediated enteropathy triggered by ingestion of gluten in genetically predisposed individuals. It is characterized by small intestinal mucosal inflammation and a wide spectrum of clinical manifestations ranging from gastrointestinal symptoms such as diarrhea, weight loss, and abdominal pain to extra-intestinal features including anemia, osteoporosis, and neurological disturbances.

The gluten-free diet (GFD) represents the cornerstone of treatment for CeD, leading to symptomatic remission and mucosal healing in the majority of patients. However, a significant clinical challenge arises in a subset of individuals, namely, those who exhibit an inadequate response to this dietary therapy. Non-responsive celiac disease (NRCD) is a broad term encompassing patients who continue to experience persistent symptoms and/or demonstrate incomplete histological recovery of the small intestinal mucosa despite maintaining a supposedly strict GFD for a period of at least 12 months [1,2]. However, many experts contest the term, viewing NRCD not as a distinct entity, but as a clinical prompt to identify a specific underlying cause.

The etiologies of NRCD are heterogeneous, but the most common cause remains inadvertent or intentional gluten exposure, highlighting the immense difficulty of maintaining a truly strict GFD. A smaller, yet critically important, subset of patients is associated with refractory celiac disease (RCD) [3,4,5,6].

RCD is specifically defined by the persistence or recurrence of symptoms and villous atrophy after 12–24 months on a GFD, in conjunction with negative CeD-specific serology [1,2,7,8,9,10,11].

The precise prevalence of NRCD is difficult to ascertain, with estimates ranging from 10% to 30%, influenced by definitions and clinical settings. A recent large, multicenter study underscored the clinical significance of this issue, finding that persistent villous atrophy—a key objective marker of non-response—was present in over 40% of adults with CeD [12]. The prevalence of true RCD is much lower (<5% of CeD patients), but it carries a grave prognosis, particularly for RCD type II (RCD-II), which is considered a pre-lymphomatous state [13].

Diagnosing and managing these conditions is profoundly challenging. NRCD requires a systematic, stepwise approach to distinguish the common causes from the rare but serious RCD. This process is complicated by the unreliability of self-reported dietary adherence and the poor correlations between symptoms, serology, and mucosal healing. The critical distinction between RCD-I (with a normal lymphocyte phenotype) and RCD-II (defined by an aberrant, clonal lymphocyte population) can only be made in specialist centers using advanced techniques like flow cytometry and T-cell clonality analysis [14,15]. This distinction is paramount, as it dictates vastly different treatment strategies and prognoses.

Furthermore, misclassification may lead to unnecessary anxiety, inappropriate therapies, or delayed diagnoses of serious complications.

Given these complexities, there is a pressing need for clear, evidence-based guidance. This review aims to synthesize the current understanding of NRCD and RCD in adults, focusing on a practical, hierarchical diagnostic framework and emerging management strategies. We will clarify the diagnostic pitfalls, highlight the central roles of dietitian-led assessment and novel biomarkers like gluten immunogenic peptides (GIPs), and discuss the therapeutic landscape for RCD, ultimately providing an actionable algorithm for clinicians. Given the clinical importance of persistent symptoms in treated CeD, distinguishing between NRCD and RCD is critical.

## 2. Methods

A narrative review of the literature was conducted. A targeted search in PubMed, Embase, Cochrane Library, Web of Science, and Scopus was performed seeking peer-reviewed studies, reviews, and conference abstracts reporting on adults with NRCD or RCD. The focus was on synthesizing current evidence to provide a practical, clinical framework for diagnosis and management.

## 3. The Standard of Care: Dietary Management and Follow-Up in CeD

### 3.1. Dietary Management

The only effective treatment for CeD is a strict, lifelong GFD, which involves the complete exclusion of gluten-containing grains such as wheat, rye, and barley. However, practical implementation of the GFD can be challenging and requires more than simply avoiding obvious sources of gluten. Hidden gluten in medications, processed foods, and supplements—as well as issues like cross-contamination and food labelling interpretation—pose ongoing challenges for patients.

Given the complexity and life-altering nature of the GFD, patients should be referred to an experienced dietitian for personalized dietary advice based on their social, cultural, and economic background [16,17]. The first and subsequent consultations should include the following elements:

1. The dietitian’s explanation of the CeD diagnosis and comorbidities, stressing the need for a strict GFD. It is important to inform patients that CeD is a chronic condition and cannot be cured, but it can be brought into remission with a strict GFD.
2. An explanation of gluten and gluten-containing grains (wheat, barley, rye), emphasizing that practically no safe level of gluten exists for patients with CeD, and a distinction between unsafe gluten-contaminated oats and safe gluten-free oats [18,19,20,21,22].
3. Guidance on reading food labels, identifying labelled gluten-free substitutes and processed foods that by their nature do not contain gluten—even if not labelled gluten-free [23].
4. Advice on cross-contamination prevention in shared kitchens, tips to reduce gluten exposure when eating out, and also tips for safe travel abroad [24,25,26,27].
5. Highlighting of the roles of patient organizations and their valuable contribution to the management of CeD and support of patients with CeD [28].

Following the initial visit post-diagnosis, dietitians play a key role in the ongoing management of CeD. This follow-up enables assessment of dietary adherence and nutritional adequacy, supports the identification of gluten exposure or nutritional imbalances, and allows for tailored education and modification of the GFD. Education should address the complexity of the GFD, promote healthy eating habits, support the maintenance of a healthy weight, and help prevent disordered eating or excessive dietary hypervigilance [29,30,31].

Through detailed dietary history-taking and targeted questioning, a CeD specialist dietitian conducts a systematic evaluation of GFD adherence and overall nutritional adequacy. This assessment, together with serology and histology data and consideration of the individual’s dietary habits, social environment, and understanding of the diet, has been shown to be superior to the standardized tools. Specialist dietetic input is more effective in identifying dietary transgressions and guiding dietary modifications than are questionnaires such as the Celiac Dietary Adherence Test (CDAT) [32,33].

Despite the compelling evidence for their efficacy, access to specialist CeD dietitians remains inconsistent and often inadequate. Systemic barriers include a lack of dedicated clinics, insufficient time allocated for the complex task of dietary counseling, and poor integration of dietetic services within gastroenterology departments [34,35]. Furthermore, under-referral by physicians and underutilization of existing services significantly contribute to suboptimal patient care. Addressing these gaps through increased investment, the development of clear guidelines on dietetic staffing, and the better integration of dietitians into core CeD-care teams is essential to delivering equitable, effective, and efficient management for all patients with CeD [36,37].

### 3.2. The Nutritional Status at the Diagnosis of CeD in Adults

The treating physician, and also the dietitian, use a combination of clinical and laboratory methods to assess the nutritional status of adults with CeD and identify specific nutrient deficiencies.

The key components of the assessment of nutritional status include the following:

1. Dietary history, specifically addressing symptoms of vitamin deficiencies and presence of other autoimmune disorders affecting nutrition; Assessment of daily intake of foods and beverages; Food Questionnaire, and an evaluation of the intake of key nutrients potentially deficient in CeD (iron, calcium, fiber, and vitamins D and B12) [38].
2. Assessment for signs of malnutrition and nutrient deficiencies; Assessments of body weight and body mass index (BMI), determining the current weight and comparing it with previous weights to evaluate weight loss.
3. Laboratory tests to check for anemia, vitamin levels, minerals, and electrolytes, as well as liver function tests and tests of serum albumin and, if necessary, exocrine pancreatic function.

### 3.3. Factors That Predispose to Non-Adherence to GFD

The reported rates for strict adherence to GFD range from 42% to 91%, depending on definition and method of assessment [39].

There are several factors recognized as being associated with lower dietary adherence to a GFD, such as: diagnosis at young age or adolescence, lower socioeconomic status, local food cultures, frequent travelling and eating in restaurants, the lack of significant symptoms at presentation, and low degree of knowledge or motivation [40,41]. Further, financial factors play a role as the gluten-free food remains expensive [42].

It is important to identify patients at risk of non-adherence to diet; this helps to develop personalized approaches for improving the degree of adherence. Targeting patients at risk of poor adherence to a GFD and increasing the number of consultations at a referral center can increased the degree of adherence in the long term [43].

### 3.4. Principles of the Follow-Up for CeD in Adults

#### 3.4.1. Importance and Goals of Long-Term Follow-Up

For patients with CeD, a lifelong GFD is not a voluntary lifestyle choice, but a medical necessity. Long-term follow-up is crucial, as persistent symptoms and mucosal changes occur in 20–40% of adult patients [11,44]. While a GFD is expected to alleviate symptoms and normalize the biochemical, serological, and histological biomarkers that were abnormal at diagnosis, neither symptomatic improvement nor serological testing reliably predicts mucosal healing [45,46].

Therefore, the primary goals of follow-up are to achieve a good quality of life (QoL), ensure symptom resolution, and confirm mucosal healing—defined as the complete normalization of the intestinal mucosa with recovery of villous architecture, absence of intraepithelial lymphocytosis, and resolution of crypt hyperplasia (Marsh 0-I stages) [47,48].

#### 3.4.2. Benefits of Structured Follow-Up

Regular follow-up with a CeD specialist improves dietary adherence [49,50,51] and provides a valuable opportunity to manage associated autoimmune disorders, address complications like bone disease, and identify warning signs suggesting the development of RCD or malignancy [50]. Patients should also be encouraged to join national CeD societies for additional support.

#### 3.4.3. Models of Follow-Up Care

There is currently no universal consensus on who should be responsible for the long-term care of adults with CeD [50].

Traditionally, follow-up has been coordinated through hospital-based outpatient clinics. In recent years, dedicated CeD centers have been established in several countries to provide specialized care. However, these centers often lack the capacity to meet the growing demand associated with the increasing prevalence of CeD.

Effective alternative models include the following:

- *Dietitian-led visits*, with physician oversight when needed, which have proven effective and cost-efficient [52,53].
- *Structured group education and on-demand consultations* [52,53].
- *Primary care management*: In countries with high levels of CeD prevalence, general practitioners (GPs) can manage long-term follow-up, though this requires adequate training and access to specialist consultation [49].
- *Telemedicine*: This is particularly well-suited for stable patients and is useful for remote monitoring, dietary support, and managing symptoms [54,55].

#### 3.4.4. Follow-Up Strategies

In general, there are two main strategies that can be employed:

A. Fixed-Interval Approach

This strategy provides a standardized framework for consistent monitoring. The optimal interval remains unclear and has not been systematically studied [50,56]. This approach is simple and consistent but may not reflect individual risk or needs. A typical regimen might involve the following:

All adult CeD patients (in the first year after diagnosis)

- Frequency: Every 3–4 months;
- Who: Gastroenterologist or internist with CeD expertise + dietitian;
- Modality: Preferably, in-person clinic visits;
- Focus on
  ∘ Disease insight and patient education;
  ∘ Reinforcement of GFD adherence;
  ∘ Correction of nutritional deficiencies (iron, folate, vitamin B12, and vitamin D);
  ∘ Screening for associated conditions (esp. thyroid disease);
  ∘ Repeated CeD serology until normalization.

Stable patients (after first year)

- Frequency: Every 1–2 years;
- Who: Shared care between primary care and specialist clinic; dietitian input as needed
- Modalities can include
  ∘ Telephone or telemedicine, which are acceptable for stable, adherent, asymptomatic patients; in-person visits if needed;
- Focus on
  ∘ Monitoring of dietary adherence (questionnaires, GIP testing if indicated);
  ∘ Periodic serology and biochemistry;
  ∘ Reassessment of nutritional quality of the GFD;
  ∘ Surveillance for comorbidities/complications, bone density measurement when indicated;
  ∘ Patient-centered counseling and support.

B. Tailored (Individualized) Approach

This patient-centered strategy is both reasonable and practical, adjusting the follow-up based on individual needs and risk profiles.

The suggested schema depending on risk profile and individual needs and preferences is as follows:

- Persistent symptoms/nutritional deficiencies: These require frequent visits and additional testing as indicated.
- Positive or persistently elevated serology: This requires closer monitoring and an assessment of GFD adherence (dietary review, GIP testing).
- High risk of non-adherence (e.g., adolescents, limited support, or ongoing GIP positivity): This requires structured dietary counseling and follow-ups that are more frequent.
- IgA deficiency: This requires monitoring with IgG anti-TG2; consider closer follow-up due to lower serologic reliability.
- Older age/comorbidities (e.g., T1DM, thyroid disease, or cardiovascular risk): These require integrated, multidisciplinary follow-ups.
- Well-controlled, asymptomatic patients without risk factors: These patients require visits that are less frequent (e.g., every 1–2 years).

#### 3.4.5. Serological Response and Mucosal Healing

A positive IgA anti-tissue transglutaminase (IgA anti-TG2) result suggests poor dietary adherence, while a negative result does not confirm strict adherence or absence of gluten exposure. Persistently positive antibody levels predict some degree of gluten intake, though the sensitivity for detecting transgressions is low (52–57%) [57,58]. CeD-specific antibodies decline within months of starting a GFD but are not accurate markers of villous atrophy [45,46,59]. In patients with IgA deficiency, IgG anti-TG2 levels often fail to normalize despite strict adherence to the indicated diet [60,61].

#### 3.4.6. Objective Evaluation of GFD Adherence

Adherence to a GFD in patients with CeD can be evaluated using validated dietary adherence questionnaires or detection of gluten immunogenic peptides (GIPs) in urine or feces [62,63]. Fecal GIPs can detect gluten exposure for up to 4 days, whereas urinary GIPs typically reflect intake within 4 to 48 h [64]. Studies have demonstrated that up to 71% of asymptomatic patients with CeD may test positive for GIPs, indicating ongoing gluten exposure despite the absence of clinical symptoms [65].

Although standardized protocols for GIP testing have not yet been established, the method is a valuable adjunct to conventional monitoring. It provides objective, real-time assessment of dietary adherence and may be particularly useful in cases of diagnostic uncertainty or ongoing symptoms, or in patients at high risk of non-compliance [66].

#### 3.4.7. Nutritional Quality of the GFD

An unbalanced GFD, often characterized by over-reliance on processed gluten-free products, can be problematic. These products are frequently lower in fiber and certain micronutrients (e.g., B vitamins, iron) yet higher in calories, saturated fats, and simple sugars, compared to their gluten-containing counterparts, to improve palatability and texture. This nutritional profile can lead to several adverse health outcomes. Patients may experience undesirable weight gain and an increased risk of developing metabolic syndrome, characterized by abdominal obesity, hypertension, dyslipidemia, and insulin resistance. Consequently, long-term adherence to a poor-quality GFD is associated with a higher risk of cardiovascular complications.

Therefore, dietary counseling must extend beyond mere gluten exclusion. It should actively promote a healthy, nutritionally balanced diet centered on naturally gluten-free whole foods, such as fruits, vegetables, lean meats, fish, legumes, and gluten-free whole grains (e.g., quinoa, brown rice, and buckwheat). This approach ensures adequate intake of essential nutrients and dietary fiber. Furthermore, education should emphasize the importance of incorporating a physically active lifestyle to the mitigation of metabolic risks and the support of overall well-being, ensuring that the GFD serves as a pathway to comprehensive health, not just symptom control [67,68].

#### 3.4.8. Follow-Up Duodenal Biopsy

The role of routine follow-up duodenal biopsy in monitoring CeD is a subject of debate, as the current evidence is insufficient to confirm that this practice improves clinical outcomes [46,69]. Mucosal healing after initiating a GFD is often a slow and incomplete process, with some studies showing that only 30% of patients achieve healing after 3–5 years [70,71].

Consequently, there is no clear indication for the performance of routine follow-up biopsies on all patients. This has led to a shift towards a more personalized, risk-stratified approach. The evidence suggests that, for a selected group of patients, a follow-up biopsy should be considered after a few years, based on factors such as older age at diagnosis, initial disease severity, and suboptimal response to the GFD [72,73]. The decision to perform a biopsy should be made in consultation with the patient, taking their preferences and values into account.

Common indications for a follow-up duodenal biopsy include the following:

- *Persistent or worsening symptoms* and/or biochemical or laboratory evidence of malabsorption.
- *Development of new “red flag” symptoms* that raise suspicion of complications such as RCD or malignancy.
- *Seronegative CeD at diagnosis*, in which case biopsies are essential for confirming the diagnosis and monitoring the response to the GFD.
- *Risk-stratified assessment*, in the context of which a biopsy may be reasonable after 1–2 years on a GFD for adults diagnosed after the age of 45 or those with initially severe presentations (e.g., ulcerative jejunitis, severe villous atrophy), to assess for mucosal healing.
- *Patient request for reassurance* regarding mucosal healing and disease control.

### 3.5. “When to Refer to a Specialist” or “Red Flags for RCD”

Indications for referral to a celiac disease specialist are summarized in Box 1.

Box 1 **Indications for referral to a specialist.**

**Persistent or new symptoms despite a gluten-free diet, including**

- Ongoing diarrhea, weight loss, or malabsorption after 6–12 months on a GFD;
- Severe abdominal pain or recurrent vomiting.

**Laboratory or histologic abnormalities, including**

- Persistent villous atrophy despite dietary adherence;
- Ongoing iron-deficiency anemia, hypoalbuminemia, or other nutritional deficiencies;
- Unexplained abnormal liver-function tests.

**Alarm features, including**

- Unintentional weight loss >10% of baseline body weight;
- Gastrointestinal bleeding, melena, or hematochezia;
- Severe or progressive abdominal pain;
- Palpable abdominal mass or lymphadenopathy.

**Suspicion for complications, including**

- Suspected or confirmed refractory celiac disease (RCD);
- Unexplained small-bowel strictures, ulceration, or perforation;
- Features suggestive of enteropathy-associated T-cell lymphoma (EATL) or small-bowel adenocarcinoma.

**Other reasons for referral include**

- Diagnostic uncertainty or incomplete initial evaluation;
- Need for specialized dietary assessment or monitoring;
- Complex comorbid autoimmune or gastrointestinal conditions.

## 4. The Non-Responsive CeD Patient

### 4.1. Subdivisions of NRCD

Epidemiological studies suggest that between 30% and 40% of adults may fall into the category of NRCD, despite the established efficacy of the GFD. The precise prevalence figures vary considerably depending on the definitions used, duration of follow-up, and clinical setting [1,2]. The optimal timing for declaring non-response remains a subject of debate; current guidelines, largely based on expert consensus, recommend an evaluation period of 12 to 24 months on a strict GFD before investigation [10,74,75].

Many experts contest the term NRCD, viewing it not as a distinct entity, but as a clinical prompt to identify a specific underlying cause. There is preference for a more precise cause-based description. Instead of telling a patient they have “NRCD,” the goal is to tell them they have “CeD with persistent symptoms due to specific cause,” or “CeD with refractoriness,” or “CeD with concomitant IBS.” This shift in language reduces ambiguity and directly informs a management strategy.

The etiologies of NRCD are highly heterogeneous, necessitating a systematic diagnostic approach. Based on clinical, serological, histopathological, and immunological features, NRCD can be categorized using the following principal causes:

1. Ongoing gluten exposure: Persistent gluten intake, whether intentional or inadvertent, is the most frequent cause of NRCD, accounting for a substantial majority of cases [3,4,5]. Intentional non-adherence is a complex, multifactorial behavior reported in up to 40% of patients and is often influenced by poor self-efficacy, a lower symptom burden, limited knowledge of CeD, and socioeconomic factors [6,44]. Inadvertent exposure is common due to the hidden gluten in processed foods, cross-contamination, and misconceptions about the GFD. Objective verification of gluten intake is recommended and can be achieved through the detection of gluten immunogenic peptides (GIPs) in stool or urine, which offers a more reliable assessment than self-reporting alone [66].
2. Slow-Responsive CeD: This less-well-defined category includes patients who exhibit a delayed clinical and histological response to a GFD. While they ultimately respond, the recovery process is protracted, extending beyond the typical expected timeframe. Differentiating slow responders from other causes of true NRCD requires patience and continued dietary diligence before escalation to more invasive investigations.
3. RCD: This is defined as the persistence or recurrence of symptoms and villous atrophy after 12–24 months of a GFD, with normal CeD serology. It is a rare but serious complication. RCD is classified as primary (no initial response to the GFD) or secondary (relapse after a period of initial response). RCD is subcategorized based on the immunophenotype of intraepithelial lymphocytes (IELs):
  - RCD-I: Characterized by a normal, polyclonal IEL population. It is often difficult to distinguish clinically from slow-responsive CeD.
  - RCD-II: Defined by the presence of an aberrant, clonal IEL population (lacking surface CD3 and CD8, with >20% aberrant cells on flow cytometry). This form is more severe, associated with complications like ulcerative jejunitis and small-bowel stenosis, and is considered a pre-lymphomatous state due to its high risk of progression to EATL [76].

This classification of RCD into types 1 and 2 is based principally on immunological criteria and may not fully encompass the spectrum of clinical presentations. Incorporating both immunological and clinical criteria into the classification system can enhance diagnostic accuracy [14,77]. Notably, a subset of patients presents with clonal or aberrant IELs in the absence of significant clinical symptoms, biochemical abnormalities, or histological damage, raising uncertainty about their classification as RCD-II. These cases are challenging to categorize and may not warrant immediate aggressive therapy; instead, they require careful monitoring, as the clonal population can remain stable or even regress over time. Recognizing this group is crucial to avoiding overtreatment and to the promotion of personalized management strategies, highlighting the need for refined diagnostic frameworks that integrate both clinical and immunological criteria [14,78].

4. Alternative or Concurrent Diagnoses: A broad range of other conditions can mimic or coexist with CeD, causing similar symptomatology. Common culprits are discussed in Section 4.4.

### 4.2. Systematic Diagnostic Approach to NRCD

The initial step in evaluating a patient with suspected NRCD is to systematically identify the underlying cause, with ongoing gluten exposure being by far the most common etiology. It is estimated that intentional or inadvertent gluten ingestion accounts for up to 80% of cases of persistent symptoms and/or villous atrophy [12]. Therefore, the diagnostic cornerstone is a comprehensive dietary assessment conducted by an experienced CeD specialist dietitian. This evaluation should extend beyond simple questioning to include a detailed food diary, an analysis of cross-contamination risks, and a review of product labels.

Objective biomarkers are increasingly critical in complementing dietary history. While CeD serology (IgA anti-TG2) is a useful tool, its limitations must be recognized. A negative serological result does not definitively confirm strict dietary adherence or the absence of gluten exposure, as antibody levels may take 24–36 months to normalize and can miss intermittent gluten intake [12]. The detection of gluten immunogenic peptides (GIPs) in stool or urine provides a more sensitive and objective measure of recent gluten ingestion, with studies revealing that a significant proportion of patients who report strict adherence still test positive for GIPs [79,80,81].

If rigorous dietary review and GIP testing confirm strict gluten avoidance, yet symptoms or enteropathy persist, the focus must shift to investigating RCD and other alternative diagnoses. A key histological finding that guides this investigation is persistent villous atrophy, which serves as a crucial objective marker of ongoing disease activity. The prevalence of persistent villous atrophy is substantial, underscoring the importance of a thorough evaluation. Studies indicate that 43% of patients have ongoing villous atrophy 1–5 years after diagnosis, a prevalence that rises to 56% in elderly patients over 70 years of age [10]. Furthermore, research shows that 24% of patients biopsied after between 15 and 61 months on a GFD still exhibit villous atrophy, with advanced age (e.g., over 45) being a significant risk factor [12].

This stepwise approach ensures an efficient and targeted diagnostic pathway for NRCD, minimizing unnecessary procedures while effectively identifying patients with true RCD or other complications.

**Figure 1:**
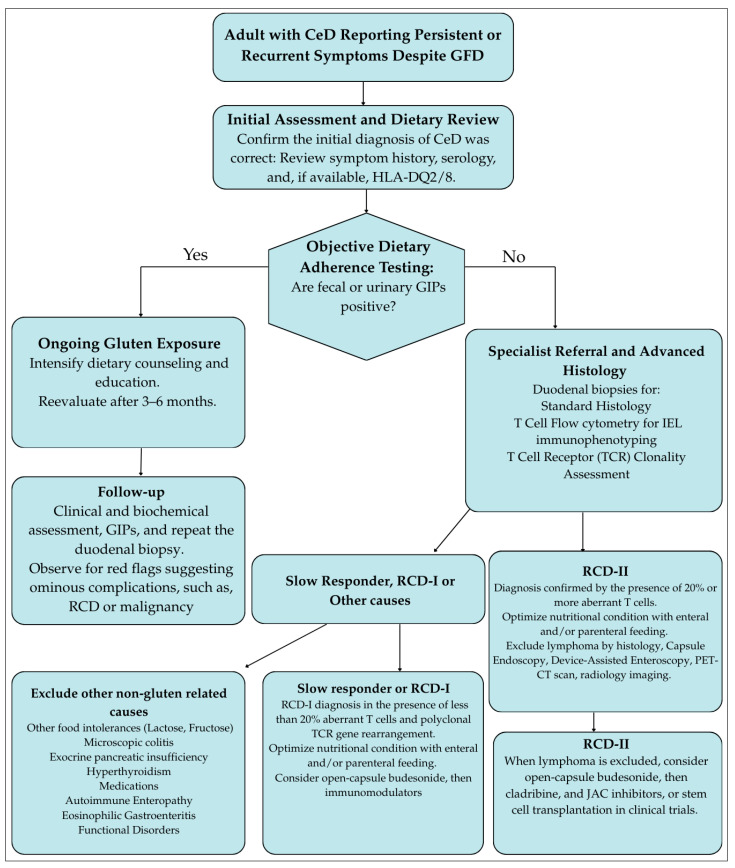
Stepwise diagnostic algorithm for the management of persistent symptoms in adult celiac disease. GFD, Gluten-free diet; JAC, Janus kinase inhibitors; NRCD, Non-responsive celiac disease; RCD, Refractory celiac disease; TCR, T-cell receptor clonality.

Figure 1 illustrates a stepwise diagnostic algorithm for the management of persistent symptoms in adult CeD.

### 4.3. The Critical Role of the Dietitian in Managing Delayed Responsiveness

In the management of NRCD, the dietitian is an indispensable member of the healthcare team, playing a pivotal role that extends far beyond basic dietary advice. For patients with delayed responsiveness, a comprehensive dietetic review is the first and most crucial investigative step, aimed at identifying persistent gluten exposure—the leading cause of NRCD—and other nutritional inadequacies. This intervention is not only therapeutic, but also diagnostic, often preventing unnecessary, invasive, and costly further investigations or premature escalation to secondary care [52].

A key challenge in CeD management is the frequent discordance between GFD adherence, symptom resolution, and mucosal healing. Asymptomatic patients may still have active disease, and symptomatic patients may have healed mucosa. In these cases of discrepant clinical indicators, the dietitian’s assessment is critical for deciding the need for a follow-up biopsy to confirm mucosal status [81,82,83]. Their expert evaluation helps determine whether ongoing symptoms are due to persistent gluten exposure, a concurrent condition, or continued inflammation despite a strict GFD. Notably, structured dietetic assessment has shown a sensitivity of 64% and a specificity of 80% in predicting ongoing villous atrophy, outperforming reliance on serology or symptom review alone [70,82].

### 4.4. Differential Diagnosis of Persistent Symptoms in Celiac Disease

A systematic approach to differential diagnosis is essential before attributing symptoms to a functional disorder or implementing further dietary restrictions. The conditions or causes that should be considered and excluded are outlined in Table 1 [84,85]:

Once these conditions have been considered and active CeD has been excluded via negative serology and confirmation of mucosal healing, a functional disorder like IBS becomes a leading diagnosis.

#### 4.4.1. Initial Misdiagnosis of Celiac Disease

A patient’s failure to improve on a GFD may not be due to NRCD, but rather because the original diagnosis of CeD was incorrect. This scenario underscores the paramount importance of confirming the CeD diagnosis before initiating a lifelong GFD. An erroneous diagnosis subjects the patient to unnecessary dietary restrictions, delays treatment for the actual underlying condition, and leads to the incorrect classification of their persistent symptoms as “NRCD.”

The diagnostic error typically arises in two ways:

1. Commencement of a GFD Prior to Diagnostic Testing:
  This is the most common reason for a diagnostic dilemma. If a patient begins a GFD before undergoing serological and histological testing, the results become unreliable. CeD-specific antibodies (IgA anti-TG2) depend on gluten consumption to be produced. A GFD will cause antibody levels to decline and potentially normalize, leading to false-negative results. On the other hand, the mucosal histological lesions (villous atrophy, intraepithelial lymphocytosis) will begin to heal on a GFD, making a biopsy inconclusive or normal, even if the patient has CeD.
2. Misinterpretation of Serological or Histological Findings:
  A diagnosis might be incorrectly assigned based on incomplete or misinterpreted data, e.g., in cases of isolated borderline positive serology without biopsy confirmation or non-specific histology without positive serology, i.e., “celiac disease mimics.” Several disorders can cause similar histological changes, and/or symptoms, leading to potential misdiagnosis: autoimmune enteropathy, common variable immunodeficiency (CVID), tropical sprue, food allergies (e.g., cow’s milk, soy, and fish), and medication-induced enteropathy. Certain drugs like olmesartan, NSAIDs, and mycophenolate mofetil can cause severe villous atrophy and symptoms indistinguishable from CeD [86,87,88].

When a patient on a GFD continues to have symptoms and an initial misdiagnosis is suspected, a systematic re-evaluation is necessary.

1. Review the original diagnostic evidence: Scrutinize the initial serology and biopsy reports. Was the serology strongly positive? Was the biopsy Marsh-III classification? Was the HLA-DQ2/DQ8 haplotype determined?
2. Perform a gluten challenge: The definitive, though challenging, method used to confirm or rule out CeD is a supervised gluten challenge. This involves reintroducing gluten into the diet (typically 3–10 g of gluten daily for 6–8 weeks) followed by repeat serological testing and duodenal biopsy. The emergence of symptoms, a rise in antibody titers, and the return of villous atrophy confirm CeD. The absence of these changes suggests an alternative diagnosis [89].
3. Investigate for “mimics”: Based on clinical suspicion, initiate a workup for other conditions.

#### 4.4.2. The Role of the Low-FODMAP Diet

For patients with IBS-type symptoms (e.g., bloating, abdominal pain, and altered bowel habits) despite a well-controlled GFD, a trial of a low-fermentable oligo-, di-, and mono-saccharides and polyols (low-FODMAP) diet may be beneficial [90,91,92,93]. This evidence-based intervention for IBS has been shown in trials and a meta-analysis to significantly reduce GI symptoms in a subset of CeD patients when implemented for a minimum of four weeks [94]. However, this diet is complex and highly restrictive. It should only be initiated after histological confirmation of remission of gluten-related inflammation, and after excluding other organic causes. Furthermore, it must be supervised by a dietitian who is expert in both gluten-free and low-FODMAP protocols to mitigate the risks of nutritional inadequacy, disordered eating, and increased food anxiety. A structured reintroduction phase is critical in order to identify specific triggers and avoid unnecessary long-term restrictions [92].

#### 4.4.3. Exocrine Pancreatic Insufficiency (EPI)

EPI is a frequently overlooked, yet treatable, cause of persistent symptoms. The prevalence of EPI is high in both newly diagnosed CeD (up to 26%) and, notably, in those with ongoing symptoms despite a GFD (28%), compared to asymptomatic patients (3%) [95]. The mechanism is likely related to previous enterocyte damage impairing the secretion of hormones that stimulate pancreatic enzyme release. The diagnosis is confirmed through non-invasive tests like fecal elastase-1. Pancreatic enzyme replacement therapy (PERT) is highly effective, and leads to improved control of symptoms like steatorrhea and bloating, enhanced nutrient absorption, and better overall quality of life [96]. Although not commonly required, PERT is a crucial consideration in confirmed cases of EPI.

## 5. Refractory Celiac Disease

### 5.1. Diagnosis of RCD

RCD is a serious complication requiring management in specialized referral centers. The diagnostic workup is multifaceted and must be rigorous, aiming to (1) reaffirm the initial CeD diagnosis, (2) definitively exclude ongoing gluten exposure through expert dietary assessment, (3) rule out all alternative causes of symptoms, and (4) conclusively distinguish between RCD types while excluding overt EATL. The diagnosis hinges on a combination of clinical, serological, endoscopic, histological, immunophenotypic, and radiological investigations [7,97].

(a) Clinical Assessment:

RCD typically manifests in adults, often years after a CeD diagnosis, with severe symptoms indicative of progressive malabsorption and systemic involvement. Key clinical features include profound chronic diarrhea, significant weight loss, abdominal pain, and features of malnutrition. Systemic signs, such as low-grade fever or symptoms of protein-losing enteropathy (e.g., peripheral edema), may be present [15,98]. In severe cases with extensive mucosal damage, patients can develop a “functional” short bowel syndrome—characterized by intestinal failure despite anatomically normal length—that may be complicated by D-lactic acidosis due to carbohydrate malabsorption and bacterial overgrowth [99,100]. Notably, a presentation of severe symptoms and extensive villous atrophy at initial CeD diagnosis should also raise immediate suspicion for RCD. Rarely, extra-intestinal manifestations may occur due to migration of aberrant T-cells to sites like the skin, lungs, or central nervous system, leading to atypical symptoms [101,102,103].

(b) Endoscopic and Histological Evaluation:

Upper GI endoscopy may reveal classic signs of villous atrophy (scalloping, mosaic pattern) but is often indistinguishable from active CeD. The critical step is obtaining a sufficient number of duodenal biopsies from the second part of the duodenum for histopathological examination and for immunophenotyping of IELs.

- *Histological examination* confirming persistent villous atrophy (Marsh-III) is a key finding. This evaluation must also specifically rule out two critical, prognostically significant conditions:
  ∘ Collagenous sprue, identified by a thickened subepithelial collagen band with entrapped inflammatory cells. This lesion is associated with severe CeD and RCD and carries a poor prognosis [104,105,106].
  ∘ Ulcerative jejunitis, which is suggested by the endoscopic finding of mucosal ulcers in the duodenum or jejunum. This is a highly suspicious feature for RCD-II and indicates a high risk of progression to EATL.
- *Video Capsule Endoscopy (VCE)*: VCE plays a pivotal role in assessing the entire small-bowel mucosa. Its primary value in RCD is in evaluating the extent of disease and identifying complications like ulcerative jejunitis, strictures, and suspicious mass lesions suggestive of EATL [107,108,109,110]. A positive VCE study at diagnosis showing extensive involvement is a prognostic marker associated with persistent villous atrophy and higher risk of adverse outcomes [111]. VCE is an ideal non-invasive tool for guiding subsequent DAE for targeted biopsies [108].
- *Deep Enteroscopy*: Device-assisted enteroscopy (DAE; e.g., single- or double-balloon) is essential for evaluating the jejunum, obtaining targeted biopsies from ulcers or strictures, and ruling out lymphoma in areas beyond the reach of a standard endoscope [104,105,106,107,108].

(c) Immunophenotyping and T-cell Clonality Analysis:

This is the crucial step for subtyping RCD [112].

- *Flow Cytometry*: This is the gold standard for differentiating RCD-I from RCD-II. It quantifies the population of aberrant IELs. Normal IELs and those in RCD-I are CD3+CD8+. In contrast, RCD-II is defined by a clonal population of >20% aberrant IELs that are cytoplasmic CD3ε+ but lack surface CD3, CD8, and T-cell receptors (TCR). Flow cytometry is superior to immunohistochemistry, as flow cytometry differentiates cytoplasmic from membranous CD3 expression and helps exclude other lymphoproliferative disorders [13,14,113,114,115].
- *T-cell Receptor (TCR) Clonality Analysis*: This PCR-based analysis involves assessing the TCR gene rearrangements in IELs. RCD-II is characterized by the expansion of a monoclonal or oligoclonal T-cell population. This clonal expansion is a hallmark of RCD-II and distinguishes it from RCD-I, which retains a polyclonal T-cell population [13,98,112]. In cases lacking TCR-gamma rearrangement, a clonal TCR-delta rearrangement can be identified [7,116]. This analysis can also identify the same malignant clone in extra-intestinal sites (e.g., skin, lung) [113,114,115].

(d) Genetic Analysis:

- *Germline Mutations*: Screening for mutations associated with immune dysregulation (e.g., *IL10RA*, *STAT1*) is important to rule out monogenic disorders mimicking RCD, such as autoimmune enteropathy [117].
- *Somatic Mutations*: Molecular profiling of aberrant IELs in RCD-II and EATL reveals recurrent gain-of-function mutations in the *JAK1-STAT3* pathway (in up to 80–90% of cases). Mutations in genes like *TNFAIP3/A20*, *TET2*, and *KMT2D* are also common and may explain resistance to certain therapies, providing potential targets for future treatment [118,119,120].

(e) Radiological and Nuclear Medicine Imaging:

Cross-sectional imaging is indispensable for assessing complications and staging.

- *CT/MR Enterography*: These techniques are valuable in visualizing mural and extraluminal complications. Key findings suggestive of RCD-II or EATL include cavitating mesenteric lymphadenopathy (necrotic lymph nodes); splenic atrophy; and small-bowel wall thickening, ulceration, strictures, and mass lesions [121,122,123,124,125,126].
- *PET-CT*: This is the most sensitive tool for identifying metabolically active lymphoma deposits that may be occult on other imaging modalities. It is crucial for staging and guiding the biopsy in suspected transformation to EATL [124,127].

(f) Nutritional Assessment:

Severe protein–calorie malnutrition, vitamin deficiencies, wasting, and hypoalbuminemia are common in RCD-II and EATL due to profound malabsorption and the systemic catabolic state. Nutritional status is a key prognostic indicator [14,128].

(g) Exclusion of EATL:

A definitive diagnosis of RCD can only be made after EATL has been rigorously excluded using the full arsenal of diagnostic tools: deep enteroscopy with biopsies, flow cytometry, TCR clonality analysis, and PET-CT imaging.

### 5.2. RCD-I Versus RCD-II

The major differences between the two types of RCD are summarized in Table 2.

### 5.3. Management of RCD

#### 5.3.1. Treatment of RCD-I

The management of RCD-I focuses on controlling inflammation and inducing mucosal healing. The first-line therapy involves nutritional support and corticosteroid treatment.

- *Nutritional Support*: Aggressive nutritional rehabilitation is essential. This includes enteral nutrition (e.g., elemental diets) or, in cases of severe malabsorption, parenteral nutrition to correct deficiencies and reverse catabolism.
- *Open-Capsule Budesonide*: The administration—opening the capsule and chewing the granules—facilitates early release in the proximal small bowel, targeting the site of inflammation. The evidence from open-label and retrospective studies demonstrates high efficacy, with clinical response in approximately 90% of patients and histological improvement in 83–90% [129,130]. A trial of open-capsule budesonide (9 mg/day divided into three doses of 3 mg) may be given. Patients often require long-term, low-dose maintenance therapy, due to the high relapse rate upon withdrawal.
- *Systemic Corticosteroids*: In patients with severe symptoms or those who cannot tolerate budesonide, a brief course of oral prednisone (0.5–1 mg/kg/day) can be used as a bridge to budesonide therapy.
- *Steroid-Sparing Immunosuppressants*: For steroid-dependent, refractory, or intolerant patients, the addition of azathioprine or 6-mercaptopurine is a common strategy. Combination therapy with prednisone and azathioprine for 52 weeks has induced clinical and histological remission in 80% of cases [131]. However, due to limited data, this approach requires careful monitoring for adverse effects.
- *Treatment Failure*: A lack of response to budesonide should prompt a re-evaluation of the original RCD-I diagnosis, including a review of flow cytometry and T-cell clonality to rule out RCD-II.
- *Monitoring*: Annual follow-up with duodenal biopsies, including histopathology and flow cytometry, is recommended to confirm response and monitor for clonal evolution.

#### 5.3.2. Treatment of RCD-II

RCD-II is a premalignant condition with a high risk of progression to EATL and must be managed exclusively in specialized tertiary centers with multidisciplinary expertise (gastroenterology, immunology, and hematology/oncology). Enrollment in clinical trials is strongly encouraged whenever possible.

- *Prerequisites for Treatment*: A definitive diagnosis of RCD-II must be established, and EATL must be rigorously excluded using PET-CT, deep enteroscopy, and biopsies before initiating therapy.
- *Nutritional Support*: As with RCD-I, intensive nutritional support, often including parenteral nutrition, is essential due to severe malabsorption.
- *Treatment Strategies*: The ultimate goal is to eliminate the aberrant T-cell clone and prevent progression to lymphoma. The following options should be considered, sequentially or in combination:
  1. Budesonide: Open-capsule budesonide (9 mg/day) can be used in clinically stable patients for symptomatic control and may induce response in a subset, though it is unlikely to eradicate the clone [129]. Severely ill patients may require intravenous steroids.
  2. Cladribine (2-CdA): This parenteral purine analogue is a common second-line therapy. Administered typically as an intravenous infusion (e.g., 0.12 mg/kg/day for 5 days, in repeated cycles), cladribine has demonstrated efficacy in reducing the aberrant T-cell clone and inducing clinical and histological response in a subset of patients [132,133]. However, the responses are often not durable, and the treatment is associated with significant immunosuppression, including profound lymphopenia and increased infection risk. For eligible patients who respond to cladribine, it may serve as a bridge to consolidative therapy with autologous hematopoietic stem-cell transplantation (auto-HSCT)
  3. Auto-HSCT: This intensive therapy offers the best chance for long-term remission and is considered for younger, fit patients (<65 years) who fail or relapse after cladribine. Studies report a 4-year survival of up to 66%, though treatment-related mortality is a significant concern [13,101,115,134,135,136].
  4. JAK inhibitors (e.g., tofacitinib): Given the high prevalence of activating *JAK*/*STAT* pathway mutations in RCD-II, JAK inhibitors represent a promising targeted therapy. Findings from some small studies show that the oral agent tofacitinib (at a typical dose of 5 mg twice daily) can induce significant clinical and histological improvement, although the impact on the aberrant clone itself may be limited [137,138]. Its long-term role remains under investigation. However, its use may be associated with significant risks, including serious infections, major adverse cardiovascular events, thrombosis, and malignancy, as outlined in its black-box warnings. Therefore, while tofacitinib is a promising agent, its use should currently be reserved for specialized centers, often as a bridge to other therapies or within clinical trials.
  5. Other therapies: Alemtuzumab, infliximab, and 6-thioguanine have been used in isolated cases with variable success, but are not standard treatments [139,140,141].
  6. Precaution: Azathioprine is contraindicated in RCD-II due to the potential increased risk of progression to EATL [9,131].
  7. Surgical resection: In cases with complicated ulcerative jejunitis or significant stenosis, surgical resection of the affected segment may be necessary before initiating immunosuppressive therapy, and can improve survival [76].

### 5.4. Prognoses of RCD and EATL

The prognosis of RCD is extremely variable between its subtypes and is heavily influenced by nutritional status and complications.

- RCD-I has a generally favorable prognosis, with a 5-year survival rate exceeding 90% with appropriate treatment [16].
- RCD-II carries a grave prognosis, with a 5-year survival rate of only 44–58% due to its high risk of transforming into EATL [13,16,142]. Poor prognostic factors include severe malnutrition, hypoalbuminemia, weight loss, and the development of complications such as ulcerative jejunitis, strictures, or opportunistic infections [13,128,142,143].

The prognosis for EATL is extremely poor. It often presents at an advanced stage, with complications like perforation or obstruction. Median survival is less than one year, even with aggressive treatment [143,144]. Outcomes are severely compromised by the patients’ profound malnutrition and poor performance status, which limits tolerance to combination chemotherapy [13]. Its current management involves multi-agent chemotherapy, with selected fit patients undergoing consolidative auto-HSCT, which can improve 2-year survival to 60–70%, in highly specialized centers [145].

A multidisciplinary approach is critical, from diagnosis through treatment, to optimize supportive care, nutritional status, and timely oncological intervention [146]. Early identification of RCD-II and vigilant surveillance for transformation remain the best strategies to improve outcomes.

## 6. Discussion and Conclusions

This review synthesizes the current evidence on the diagnosis and management of NRCD and RCD in adults. A central theme that emerges is the necessity of a systematic, stepped approach: beginning with the most common cause of persistent symptoms—ongoing gluten exposure—before investigating rarer complications like RCD.

The initial management of NRCD hinges on an objective evaluation of GFD adherence, as self-reporting is notoriously unreliable. The emergence of objective biomarkers like GIPs represents a significant advance, objectively identifying gluten exposure in many asymptomatic patients [64,65,83]. Looking forward, the validation of other biomarkers, such as fecal calprotectin for assessing ongoing mucosal inflammation or serum IL-2 for T-cell activation, could further refine this assessment.

Furthermore, a broad differential diagnosis, including functional disorders and exocrine pancreatic insufficiency, is also critical to ensure treatable conditions are not overlooked [92,94,95].

The differentiation between RCD-I and RCD-II is the pivotal step in the management of RCD, with profound prognostic and therapeutic implications. This involves duodenal biopsies with flow cytometry and T-cell receptor (TCR) clonality analysis to identify the aberrant, clonal intraepithelial lymphocytes characteristic of RCD-II [97,98,112]. The emerging recognition of an intermediate patient group with increased numbers of aberrant T cells and clonality but minimal symptoms underscores the need for a classification system integrating both immunological and clinical criteria to prevent overtreatment.

A critical limitation in guiding RCD therapy is the available evidence, which consists predominantly of retrospective cohorts, small open-label trials, and case series, due to the rarity of the condition. Within this constrained framework, open-capsule budesonide is a recognized first-line therapy for RCD-I [129,130,147]. The role of immunomodulators like azathioprine is less defined, but it appears beneficial in steroid-dependent or refractory cases [131]. In the management of RCD-II, while cladribine and auto-HSCT can induce remission in a subset of patients, the risk of progression to EATL remains high, and outcomes are poor [13,115,132,133,134]. These findings must be interpreted with caution given the small sample sizes and lack of controlled comparative data. The exploration of targeted therapies, particularly JAK inhibitors like tofacitinib for cases with relevant gain-of-function mutations, is promising based on preliminary data, though more robust clinical trials are clearly needed [137,138].

The prognostic disparity—with 5-year survival exceeding 90% for RCD-I but falling below 50–58% for RCD-II—highlights the imperative of early and accurate diagnosis [13,142]. Outcomes are further compromised by severe malnutrition at presentation, emphasizing that nutritional support is a fundamental component of therapy, not merely supportive care. For the unfortunate minority who progress to EATL, the prognosis remains dismal, managed with combination chemotherapy and, in selected cases, auto-HSCT [145].

In conclusion, the management of NRCD and RCD requires a high index of suspicion, a systematic diagnostic approach, and early referral to a multidisciplinary team with expertise in complex CeD. It is crucial to acknowledge that current recommendations, particularly for RCD-II, are based largely on retrospective cohorts, small open-label studies, and expert opinion, due to the rarity of the condition.

While significant challenges remain, future progress hinges on addressing specific, critical research gaps. The key priorities, summarized in Box 2, include the validation of novel biomarkers; the initiation of robust therapeutic trials; and a deeper understanding of disease progression, with the aim of enabling pre-emptive strategies. Addressing these priorities through concerted research efforts is essential for developing robust evidence-based guidelines and ultimately improving the prognoses for these challenging patient populations.

Box 2 **Critical gaps that guide future research.**

1. Standardization of Diagnostics

Prospective studies are needed to validate and establish standardized protocols for biomarkers like GIPs and serum IL-2 in routine monitoring and in the evaluation of NRCD.

2. Treatment Trials

Multicenter international registries and collaborative trials are essential to evaluate existing and new therapies for RCD-II using standardized definitions of treatment response.

3. Risk Stratification

Research is needed to understand the natural history of clonal IEL populations and to identify biomarkers that predict progression to overt RCD-II or EATL.

4. Nutritional Interventions

The impacts of specific, aggressive nutritional support regimens on treatment tolerance and survival in RCD and EATL deserve focused study.

## Figures and Tables

**Table 1:** This summarizes the differential diagnosis and outlines the first-line diagnostic tests.

*Category of Cause*	Specific Condition	Key Clinical Clues	Suggested First-Line Investigations
*Persistent Active CeD*	Ongoing gluten exposure (Intentional/Inadvertent)	Poor response despite reported GFD adherence. Lack of serological normalization.	Dietitian-led assessment, fecal/urinary GIPs, and serology (IgA anti-TG2).
	Slow-responsive celiac disease	Gradual improvement over >12–24 months, but incomplete.	Follow-up duodenal biopsy, clinical and serological monitoring.
	Refractory celiac disease (RCD)	Severe symptoms, weight loss, and malnutrition, despite strict GFD >12–24 months and negative serology.	Duodenal biopsy with IEL flow cytometry, TCR clonality, and cross-sectional imaging (CT/MR enterography).
*Alternative/Concurrent Diagnoses*	Functional disorders	Irritable bowel syndrome (IBS) [Bloating, abdominal pain, altered bowel habits after mucosal healing is confirmed].	Exclude other causes first. Trials of low-FODMAP diet (with dietitian), breath tests (lactose/fructose) if applicable.
	Other GI inflammatory/immune	Microscopic colitis (Watery, non-bloody diarrhea, weight loss).	Colonoscopy with random biopsies.
	Exocrine pancreatic insufficiency (EPI)	Bloating, steatorrhea (pale, foul-smelling stools), weight loss.	Fecal elastase-1 test.
	Eosinophilic Gastroenteritis	Diarrhea, abdominal pain, nausea. Peripheral eosinophilia may be present.	Duodenal/colonic biopsies (assess for eosinophilia), Peripheral blood eosinophil count.
	Autoimmune Enteropathy	Severe diarrhea and autoimmunity. Often seronegative.	Duodenal biopsy, specific autoantibodies (e.g., anti-enterocyte), immunoglobulin levels.
	Misdiagnosis of CeD	Other enteropathies (e.g., tropical sprue, drug-induced, HIV enteropathy).	Review initial diagnostic criteria for CeD. History of travel, medication use (e.g., Olmesartan, NSAIDs), and risk factors. Gluten challenge if pre-diet testing was incomplete, stool for infection, medication review.
	Malignancy	Enteropathy-associated T-cell lymphoma (EATL) (alarm symptoms (e.g., fever, night sweats, abdominal pain, obstruction, rapid clinical decline)).	PET-CT, deep enteroscopy with biopsies, IEL flow cytometry, TCR clonality.
		Small-bowel adenocarcinoma (Unexplained weight loss, iron-deficiency anemia, obstruction.)	Cross-sectional imaging (CT/MR enterography), video capsule endoscopy, deep enteroscopy.

Abbreviations: CeD, Celiac disease; EATL, Enteropathy-associated T-cell lymphoma; EPI, Exocrine pancreatic insufficiency; GFD, Gluten-free diet; GIPs, Gluten immunogenic peptides; HIV, Human immune deficiency virus; IBS, Irritable bowel syndrome; IgA TG2, Immunoglobulin A anti-tissue transglutaminase; IEL, Intraepithelial lymphocyte; TCR, T-cell receptor; CT, Computed tomography; MR, Magnetic resonance; FODMAP, Fermentable oligo-, di-, mono-saccharides and polyols; NSAIDs, Non-steroidal anti-inflammatory drugs; PET-CT, Positron emission tomography–computed tomography; TCR Clonality, T-cell receptor clonality.

**Table 2:** Major differences between RCD-I and RCD-II.

Feature	RCD-I	RCD-II
Malabsorption features	In ~50% of cases	In >80% of cases
Key risk factors	Older age, poor GFD adherence	Older age, HLA-DQ2 homozygosity (~60%)
CeD serology	Usually negative	Usually negative
Hypoalbuminemia	Present in ~50% of cases	Present in almost all cases
Endoscopic ulcers/stenosis	Rare	Common
Persistent villous atrophy	In all cases	In all cases
Defining diagnostic method	Flow cytometry: <20% aberrant IELs	Flow cytometry: >20% aberrant IELs and T-cell clonality
Immunophenotype (flow cytometry)	Normal (sCD3+CD8+)	Aberrant (cCD3ε+, sCD3−, CD8−, TCR−)
Immunohistochemistry	Normal IEL phenotype (CD3+CD8+)	Loss of surface CD8 on IELs
5-year survival	>80%	<50%; High risk of EATL

RCD, Refractory celiac disease; IELs, Intra-epithelial lymphocytes; TCR, T-cell receptor; EATL, Enteropathy-associated T-cell lymphoma.

## Data Availability

The data presented in this study are available upon request from the corresponding author, due to ethical concerns.
